# Social isolation and cancer management after the 2011 triple disaster in Fukushima, Japan: A case report of breast cancer with patient and provider delay

**DOI:** 10.1097/MD.0000000000004027

**Published:** 2016-07-01

**Authors:** Akihiko Ozaki, Claire Leppold, Masaharu Tsubokura, Tetsuya Tanimoto, Shigehira Saji, Shigeaki Kato, Masahiro Kami, Manabu Tsukada, Hiromichi Ohira

**Affiliations:** a Department of Surgery; b Department of Research; c Department of Radiation Protection, Minamisoma Municipal General Hospital, Minamisoma, Fukushima; d Department of Internal Medicine, Jyoban Hospital of Tokiwakai Group, Iwaki; e Department of Medical Oncology, Fukushima Medical University; f Research Institute of Innovative Medicine, Jyoban Hospital, Iwaki, Fukushima; g Medical Governance Research Institute, Minato-ku, Tokyo, Japan.

**Keywords:** disaster, Minamisoma, social support

## Abstract

Breast cancer patients may present with patient delay or experience provider delay—2 factors which can lead to a late-stage diagnosis and poor prognosis. Mass disasters drastically change social structures, and have the potential to contribute to these delays. However, there is little information available on patient and provider delay related to cancer after disasters. In March 2011, an earthquake, followed by a tsunami and nuclear accident struck Fukushima, Japan. In July 2014, a 59 year-old Japanese widow, living alone, presented to our hospital with a lump and pain in her right breast, which had originally appeared in April 2011 and continuously deteriorated for 3 years and 3 months. She was diagnosed with stage IIIB right breast cancer. Detailed history revealed that she was exposed to social isolation in the aftermath of the disasters due to evacuation of her friends and daughter. Although she regularly saw her general practitioner, she did not disclose her breast symptoms for 1 year and 5 months, at which time she was falsely diagnosed with intercostal neuralgia. She did not seek further medical attention for the breast symptoms for another 1 year and 10 months, despite multiple clinic visits for unrelated reasons. The present disasters, particularly the nuclear disaster, seem to have led to the social isolation of local residents, reducing their opportunities to discuss health concerns with others and seek subsequent medical attention.

This case highlights that social isolation may contribute to patient and provider delay in breast cancer patients, as accentuated in this disaster setting.

## 1 Introduction

Breast cancer is the most frequently diagnosed cancer and the most common cause of cancer death among females worldwide.^[1]^ Early detection and treatment are imperative for reducing the burden of breast cancer.^[1]^ Yet, the majority of breast cancer cases are identified after symptoms appear, such as a breast lump or nipple discharge.^[2]^ Moreover, 20% to 30% of symptomatic breast cancer patients present with patient delay, generally defined as an interval 3 months or longer from discovery of symptoms to first medical consultation.^[3]^ Additionally, some patients experience provider delay, similarly defined as an interval 1 month or longer from first medical consultation to beginning of treatment.^[4]^ Both of these delays can lead to late-stage diagnosis and contribute to poor prognosis, highlighting the importance of early medical consultation and intervention.^[4,5]^

Traditionally, patient delay and provider delay have been studied separately.^[4]^ In an extensive review, Khakbazan et al have identified several factors associated with patient delay, including little knowledge regarding breast cancer and symptoms other than lump.^[2,4]^ In addition, psychological factors, such as not attributing symptoms to breast cancer or distrust in health care, may be associated with delays in seeking care.^[2,3,6]^ On the other hand, provider delay is generally attributed to medical diagnostic error, possibly providing a false sense of reassurance to patients.^[4]^ In integrated assessments of both patient and provider delay, there has recently been a considerable research focus on the importance of patients’ social surroundings, particularly the availability of social support.^[2,4]^ However, previous studies have not taken into account that these social surroundings can change over time, and that changes may affect the processes of seeking help and subsequent treatments. Mass disasters, such as hurricanes, tsunamis, or earthquakes, provide a lens by which we can assess how drastically changed social structures may contribute to both patient and provider delay.^[7–11]^

**Figure 1:**
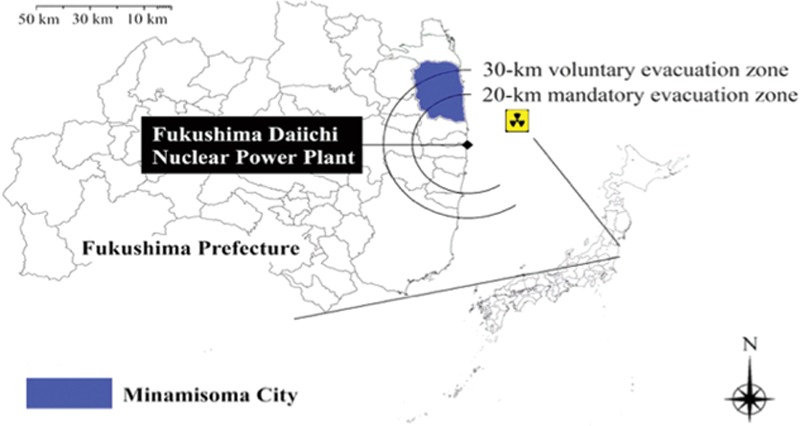
Geographical location of Minamisoma City.

In March 2011, Northeast Japan was struck by the Great East Japan Earthquake, followed by a tsunami and nuclear accident at Fukushima Daiichi Nuclear Power Plant, known as Japan's triple disaster.^[12]^ Minamisoma City, located 14 to 38 km north of the power plant (Fig. 1), was severely affected by the triple disaster. After the release of radioactive agents, those concerned about health problems related to nuclear contamination, particularly the young and middle-aged generation, evacuated from the city.^[13]^ The original population of 72,000 decreased to approximately 10,000 immediately after the disasters, gradually recovering to 45,000 in 2012.^[13]^ Additionally, those who remained in the city experienced rapid and long-lasting deterioration of local communities and lifestyle changes.^[13]^ These rapidly changed social structures may have impacted care of cancer patients. However, little investigation has been conducted on patient and provider delay pertaining to cancer after disasters, especially nuclear disasters.

We experienced a breast cancer patient with long-term patient and provider delay after the triple disaster. Detailed history revealed that she experienced social isolation in the aftermath of the disasters, which may have reduced her opportunities to discuss health concerns with others and seek subsequent medical attention.

## 2 Case presentation

In July 2014, a 59-year-old Japanese female with no significant past history presented to our hospital with a lump and pain in her right breast that had gradually worsened for 3 years and 3 months. She had never undertaken any mammography breast cancer screenings, although it was offered through the local government of Minamisoma City. In April 2011, she first noticed a lump in her right breast. It continued to gradually enlarge, and in September 2012, pain appeared in her breast, leading to the first disclosure of her symptoms to her general practitioner, whom she had frequently (3–4 times per year) visited for antimicrobial treatment for recurrent urinary tract infections since March 2010. However, she was falsely diagnosed with intercostal neuralgia due to her pain, without consideration of the lump. She did not seek a second opinion, reporting that she trusted the judgment of her doctor. From this event, she continued to see her general practitioner, while ignoring her deteriorating symptoms. She had knowledge of breast cancer, and suspected that it may have caused the lump, yet avoided thinking about it. Her general practitioner retired in December 2012, and another physician immediately took over. However, while she saw the new physician twice in 2013, she did not disclose her breast symptoms. In July 2014, 1 month after her lump abruptly enlarged, she first presented to our hospital, where postdisaster specialized breast cancer care had restarted in August 2011.

At initial examination, her lump was 2 cm in diameter, fixed to her chest wall. The pathological examination showed tumor cells of invasive ductal carcinoma, with positive estrogen receptor, negative progesterone receptor, and negative human epidermal receptor type 2. None of the further examinations revealed any lymph node swelling or distant metastasis. She was diagnosed with stage IIIB right breast cancer. Because of her intense pain, surgery was prioritized before medical therapy. In August 2014, a right mastectomy and axillary lymph node dissection was conducted. The surgery revealed that the tumor had invaded the right pectoralis major muscle, part of which was resected en bloc. The pathological diagnosis was stage IIIB right breast cancer, with negative margins. Adjuvant chemotherapy of FEC, consisting of 5-fluorouracil, epirubicin, and cyclophosphamide, for 4 cycles, followed by tri-weekly paclitaxel for 4 cycles, was administered after the surgery. In May 2016, she continues hormonal therapy with aromatase inhibitor, with no relapse or adverse effects.

Additional history taking revealed that the patient was exposed to social isolation after losing contact with her daughter and friends in the aftermath of the March 2011 disasters, which likely resulted in lessened opportunities to seek advice about her changed health status. Before the disasters, she had lived by herself since the death of her husband. Her daughter and her family, living in the same city, frequently visited her and provided social support. Additionally, she regularly kept in touch with her friends in the neighborhood. After the disasters, the patient, her daughter's family, and her friends were physically unharmed. However, the daughter's family evacuated to another city, approximately 80 km away, in fear of radiation exposure. The patient hesitated to relocate with them, eventually deciding to remain because she did not want to become a burden to them in the already difficult postdisaster period. This concern is also what led her to rarely call them on the phone after their evacuation, or contact her neighborhood friends. She did not use any social networking services.

## 3 Discussion

This is a case of advanced breast cancer diagnosed after significant patient and provider delay, and highlights that social isolation may contribute to delays, especially in disaster settings.

In general, reasons for patient delay include little knowledge about breast cancer, symptoms other than lumps, not attributing symptoms to breast cancer, distrust in health care, and poor access to health care; however, these factors did not seem to be present in this case.^[2–4,6]^ The patient reports having knowledge of breast cancer and suspecting that it was the cause of her symptoms. Although she never undertook breast cancer screenings, this does not necessarily represent a lack of knowledge as it is reported that only 30% of women in Japan attend any kind of breast cancer screening programs.^[14]^ The patient regularly (3–4 times per year) saw her general practitioner for recurring urinary tract infections up until he retired in December 2012, at which point she began seeing the physician who replaced him semiregularly (2 times a year). She additionally states that she had no distrust towards health care even after a misdiagnosis. It is difficult to conclusively decipher the cause of her behaviors to not seek care for the breast symptoms; yet, considering that she experienced drastic changes to her social life after the disasters, it seems reasonable to hypothesize that social isolation could have been the main contributor to her patient delay. Social networks are generally the first place where patients seek advice to make sense of their symptoms, and these interactions can influence their further actions.^[2,4]^ Therefore, it can be thought that loss of social connections has a negative impact on the process of deciding to seek medical consultation, as it seems to have happened in this case.

Social isolation also increases the risk of experiencing provider delay, a type of delay that has traditionally been attributed to the actions and capacities of healthcare providers.^[4]^ Although the misdiagnosis is a notable part of this case, it is additionally surprising that the patient had been in frequent contact with her general practitioner for the first 1 year and 5 months after noticing symptoms without disclosing them, and did not seek care for the 1 year and 10 months after the misdiagnosis, despite symptom deterioration. This suggests that whereas lack of access to healthcare or infrequent visits can contribute to patient or provider delay,^[2,4,15]^ regular visits may not prevent it. In this regard, the disaster, which occurred the month before she first noticed her symptoms, may have cast an influence over this entire process, specifically through triggering her social isolation—a factor likely to have contributed to both the initial delay in disclosing symptoms, and subsequent delay in seeking further medical attention after the misdiagnosis. Another possible explanation for this may be tied to the phenomenon of normalcy bias, defined as a state of mind which leads people to underestimate possible risks, assuming that adverse outcomes may happen to others, but not themselves.^[16]^ In the present case, the patient may have unconsciously avoided thinking of the consequences of her actions, despite consciously acknowledging a potential link with breast cancer. Previous studies suggest that a loss of opportunities to discuss personal problems, due to social isolation, may worsen normalcy bias,^[16,17]^ and this may have been a factor related to the long-term delay in the present case.

The disaster setting where this case occurred can be further discussed in reference to findings from previous disasters. It has been suggested that cancer patients with strong social networks were more likely to reach medical care compared with those with poor social networks after Hurricane Katrina.^[8,18]^ Minamisoma City was severely affected by 2 natural disasters (earthquake, tsunami) and a subsequent nuclear disaster. It is estimated that completely decommissioning the reactors of Fukushima Daiichi Nuclear Power Plant will take 30 to 40 years, which may postpone disaster recovery while causing prolonged anxiety and concern among local residents.^[19]^ We underline that there are potential differences in the impacts of nuclear disasters and natural disasters to local communities. Natural disasters mainly lead to physical and social loss via the death of family members or friends.^[20,21]^ Communication networks and traffic are disrupted, and those who lose their homes are displaced to shelters, temporary housing, or new residences.^[21–23]^ Nevertheless, it has been shown that majority of areas struck by natural disasters are eventually able to return to their original conditions, in terms of the economy and population.^[24,25]^

On the other hand, in nuclear disasters, long-term psychosocial impacts may be more severe than physical loss.^[26,27]^ Fear of radiation is a particularly strong component in long-term psychosocial impacts,^[20,27]^ as radiation exposure is commonly known to be dangerous,^[28]^ yet it is invisible and finding accurate information on radiation risk may be difficult.^[20,27,29]^ Evacuation, which can bring about the biggest social impacts related to nuclear disasters, may be strongly motivated by this fear.^[27]^ It can be difficult for evacuees to judge if or when to return, often resulting in long-term displacement, which may delay recovery of damaged areas.^[30]^ Residents who remain in contaminated areas also face adverse effects: to avoid radiation exposure, they may avoid going outside, stay at home all day, and rarely socialize.^[20,29]^ Conflicting perceptions of radiation risk can result in discordance of families and communities, and disparities in governmental restrictions and compensation may additionally contribute to community tension.^[26,29]^ Therefore, it can be argued that nuclear disasters have a larger potential to lead to long-term social isolation, as compared with natural disasters.

This case highlights the influence of social isolation to the process of seeking care, and presents several lessons we can learn from the progression of her treatment. The fact that the 1 year and 5 months of delay before disclosing symptoms occurred while the patient was frequently visiting a physician, and that she sought no further medical consultation for the breast symptoms in the 1 year and 10 months after her misdiagnosis, may be difficult for physicians to interpret; the accurate diagnosis and treatment of patients is a daunting goal when they do not disclose their symptoms or seek medical attention. Apart from the misdiagnosis, it is difficult to propose areas for physician-level improvements that could have led to the better management of this patient. Yet, this case serves as a vivid example of how health may be affected by a range of factors outside the doctor's office, and we hope it may lead to improved physician awareness of the potential for delay, particularly in patients facing significant social changes such as isolation or loss of contact with loved ones. Fortunately, after presentation to our hospital, the patient underwent surgery, chemotherapy, and hormonal therapy. Considering her original long-term reluctance to seek medical care, this fact is notable, as it has been suggested that social support is important for the continuation of treatment.^[4,31]^ We believe that regular visits to medical institutions during breast cancer treatment may have provided an alternative form of support for the patient, possibly mitigating some effects of her social isolation. A previous study suggests that regular hospital visits are associated with control of chronic diseases in disaster settings.^[32]^ A possible reason for this is that regular hospital visits can provide patients with increased opportunities to talk about health problems, and for doctors to empower them.^[32]^ In irregular situations, such as the aftermath of disasters, frequent clinical visits may count more, as compared with routine settings, in the management of cancer patients.

In conclusion, we experienced a case of advanced breast cancer with significant patient delay and provider delay, possibly induced by social isolation after the disasters. The 2011 triple disaster, particularly the nuclear disaster, is likely to have led to the patient's social isolation through rapid social changes. This isolation could have subsequently affected her delays, and late-stage diagnosis of breast cancer. This case highlights that social isolation can contribute to delays, and delays may be more frequent in context of changing social environments, such as disasters. Nevertheless, this case also suggests that, after a diagnosis, healthcare providers may be able to mitigate some effects of social isolation during the process of regular hospital visits. These visits may lead to increased opportunities for patients to talk about their health concerns, and for doctors to empower them. This presents a path for cancer patient support that clinicians should be aware of, in both disaster and nondisaster settings.

## Acknowledgments

We would like to thank Mr Shuhei Nomura, and Dr Mutsuko Ohnishi, for their useful opinions on this study.
